# Acupuncture for premature ventricular complexes without ischemic or structural heart diseases: A systematic review and meta-analysis of clinical and pre-clinical evidence

**DOI:** 10.3389/fmed.2022.1019051

**Published:** 2022-12-08

**Authors:** Yiyi Cai, Claire Shuiqing Zhang, Shaonan Liu, Li Zhou, Boyan Tang, Wensheng Chen

**Affiliations:** ^1^State Key Laboratory of Dampness Syndrome of Chinese Medicine, The Second Affiliated Hospital of Guangzhou University of Chinese Medicine, Guangdong Provincial Hospital of Chinese Medicine, The Second Clinical College of Guangzhou University of Chinese Medicine, Guangzhou, China; ^2^Guangdong Provincial Academy of Chinese Medical Sciences, Guangzhou, China; ^3^School of Health and Biomedical Sciences, STEM College, RMIT University, Melbourne, VIC, Australia

**Keywords:** acupuncture, ventricular premature complexes, randomized controlled trial, systematic review, meta-analysis

## Abstract

**Background:**

With increasing evidence suggesting potential benefits, acupuncture is often applied to the treatment of premature ventricular complexes (PVCs), particularly in symptomatic patients who fail or are unsuitable for medications or refuse catheter ablation. However, the existing clinical evidence is inconsistent.

**Objectives:**

This review aims to systematically evaluate the effectiveness and safety of acupuncture therapies for PVCs without ischemic or structural heart diseases, when it is compared with sham/placebo acupuncture or usual care, or used as an add-on therapy to routine care; and to summarize existing pre-clinical research evidence supporting the effects of acupuncture therapies for this clinical condition.

**Methods:**

Four English-language databases, four Chinese-language databases and seven clinical registries were searched from their inceptions to May 21, 2021 and updated to November 01, 2022. Trials comparing acupuncture with sham acupuncture or evaluating the add-on effects of acupuncture were included. Primary outcomes are the number of premature ventricular beats (PVBs) and effective rate defined as “the proportion of participants with over 50% decrease in the number of PVBs from baseline to the end of treatment measured by 24-h Holter”.

**Results:**

A total of 479 records were identified with nine trials involving 847 participants included in this review. Meta-analysis on two sham-control trials with low risk of bias for all domains suggested that acupuncture could significantly reduce the number of PVBs (RR 3.83, 95% CI [2.19, 6.7], *I*^2^ = 0%). Moreover, the combination of acupuncture and standard treatment was superior to standard treatment alone in reducing the burden of PVBs (RR 1.21, 95% CI [1.08, 1.36], *I*^2^ = 0%). Though no treatment protocol consensus was announced, body acupuncture on point PC6, HT7, DU10, DU11, and ST36 with duration of needle retention ranging from 15 to 30 min for a 4-week treatment period is broadly used by the included trials. For experimental evidence, five studies explored the mechanisms of acupuncture for PVCs were eventually included into analysis and PC6 was the most frequently studied acupuncture point. Moreover, a reduction of electrical activity of sympathetic nerves in experimental animals undergoing electro-acupuncture was observed by four of these studies.

**Conclusion:**

Sham-controlled RCT evidence with moderate-level certainty suggested that acupuncture could be a therapeutic option to reduce the burden of PVBs in patients without ischemic or structural heart diseases. Further clinical studies using validated and reliable outcome measurement instruments and bench research to unveil the mechanisms of acupuncture stimulation and point-specific effects for PVCs are needed.

**Systematic review registration:**

[https://www.crd.york.ac.uk/PROSPERO/display_record.php?RecordID=262132], identifier [CRD42021262132].

## Introduction

Premature ventricular complexes (PVCs) are commonly detected arrhythmias in general population (1). The prevalence of PVCs was reported between 1 and 6% on a standard 12-lead electrocardiography and around 40–75% by 24–48 h Holter monitor with an increasing trend with age (1–7). PVCs can be asymptomatic or present symptoms of palpitation, dizziness, chest pain, fatigue, dyspnea, and even presyncope (5). PVCs are usually benign in individuals without confirmed ischemic or structural heart diseases. Structural heart diseases are defined as structural abnormalities of the heart detected through currently available tests or examinations, including echocardiography, Magnetic Resonance Imaging, exercise stress testing, endocardia biopsy and other existing objective tests, as well as autopsy. Recent evidence suggested that PVCs are associated with increased risks of almost all heart diseases, although the underlying mechanisms are yet to be confirmed (4). For ischemic or structural heart diseases, anti-arrhythmia is not considered as the primary managements. While for PVCs patients without ischemic or structural heart diseases, to alleviate symptom and improve quality of life are the key reasons for seeking clinical management even though they are at relatively low risk of sudden cardiac death (2, 3, 6). Therefore, reducing the number of premature ventricular beats (PVBs) is beneficial, particularly for those suffering over 10,000–15,000 PVBs each day (7).

Standard treatment for PVCs mainly consists of anti-arrhythmic medications such as beta-blockers, non-dihydropyridine calcium channel blockers (1); for severe PVCs cases that are either symptomatic or likely responsible for systolic dysfunction, or patients who are intolerant or do not respond well to medications, catheter ablation should be considered (1, 3, 5, 8, 9). These mainstream treatments for PVCs are facing the following challenges: (1) anti-arrhythmic medications do not provide symptom relief to all patients undertaking anti-arrhythmic medications (10); (2) catheter ablation is an invasive procedure which may eradicate PVCs, but recurrence or worsening of PVCs after the procedure are often observed in clinical practice (11); (3) catheter ablation is generally considered and recommended in PVCs originated from a right ventricular outflow tract. However, catheter ablation for non-right ventricular outflow tract originated PVCs is more challenging due to anatomical obstacles (2, 10, 12–14). Therefore, although both medical treatment and catheter ablation are recommended as the first-line therapies for symptomatic PVCs, clinical decisions are usually made based on patients’ preference (5, 15).

Acupuncture is a key component of Chinese medicine therapies that involves the insertion of very thin needles through skin at specific points. Moderate or high certainty evidence was found in the areas of physical function improvement and pain relief by acupuncture (16–19). According to experts’ consensus, acupuncture was recommended as an effective adjunctive therapy to regulate heartbeat rhythm for patients with arrhythmia (20). Although the number of clinical trials on acupuncture for cardiac arrhythmia increased in recent years, the results were inconsistence and inconclusive (21–23). In light of the growing number of clinical trials evaluating the effects of acupuncture therapies published in recent years, we conducted this systematic review focusing on the effectiveness and safety of acupuncture therapies for PVCs. The key research questions of this systematic review is: (1) In PVCs patients without ischemic or structural heart diseases, compared to sham or placebo acupuncture or usual care, is acupuncture therapies safe and effective in reducing the burden of PVBs? (2) Is adding acupuncture therapies to routine care safe and effective in reducing the burden of PVBs for PVC patients without ischemic or structural heart disease?

## Methods

### Database search

Four English-language databases, four Chinese-language databases and six online clinical trial registration platforms were searched from their inceptions to May 21, 2021 and updated to November 01, 2022 (Supplementary Table 1). These are: PubMed, EMBASE, Cochrane Library, Cumulative Index to Nursing and Allied Health Literature; Chinese Biomedical Database, Chinese National Knowledge Infrastructure, Chongqing VIP Database, Wanfang Database; the Chinese Clinical Trial Register,^1^ Australian New Zealand Clinical Trials Registry,^2^ Clinical Research Information Service, Republic of Korea,^3^ EU Clinical Trials Register,^4^ Japan Primary Registries Network,^5^ and ClinicalTrial.gov.^6^

**FIGURE 1:**
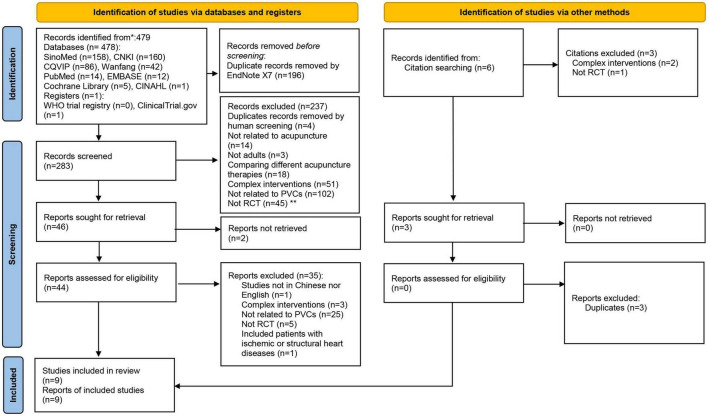
PRISMA 2020 flow diagram.

Search strategies consist of two groups of terms: PVCs and acupuncture. The procedure of search and screening is presented in Figure 1. Reference lists of relevant RCTs and systematic reviews were screened to identify additional trials and recorded under the category of “identification of studies *via* other methods.” Search strategy for PubMed and Chinese Biomedical Database are presented in Supplementary Table 1 as an example.

### Study selection and screening

Selection criteria for eligibility were established and confirmed through discussion within the research team. Studies were screened independently by two reviewers (YC and CZ). Disagreements between these two reviewers were resolved by discussing with a third author (SL).

Studies were included if they: (1) recruited patients aged over 18 years and diagnosed with PVCs without ischemic or structural heart diseases; (2) compared the effects of acupuncture therapies (including manual acupuncture, electro-acupuncture, acupressure, auricular acupressure, and auricular acupuncture) with sham or placebo acupuncture, or standard therapies recommended by clinical guidelines (including suggestions on life style modifications, anti-arrhythmic medications); or evaluated the add-on effects of acupuncture therapies to standard therapies; (3) reported at least one of the following outcome measures, either the primary or secondary or safety outcome measures.

Primary outcome measures: the number of PVBs measured by 24-h Holter; effective rate defined as the proportion of participants with over 50% decrease of PVBs from baseline to end of treatment measured by 24-h Holter;

Secondary outcome measures: scores or scales of symptoms improvement (such as a 10-scale Visual Analogue Scale), quality of life (such as SF-36);

Safety outcome: adverse events.

Studies were excluded if they: (1) included participants diagnosed with other arrhythmia (such as arterial fibrillation, supraventricular tachycardia) or severe cardiovascular conditions (ischemic, valvular, hypertensive and congenital heart disease, aneurysm, heart failure, etc.); (2) adopted complex treatment that could not specify the effects of acupuncture therapies (e.g., acupuncture plus Chinese herbal medicine plus metoprolol vs. metoprolol); (3) compared different types of acupuncture therapies (e.g., acupuncture vs. acupressure); (4) quasi-RCTs which allocated participants based on quasi-randomization methods (e.g., the order of patients attending the clinics, patients’ birthday, or case record numbers), such a design is likely to cause selection bias. Since a core outcome set has not been established for clinical trials of PVCs, the outcome domains for this review was determined through discussion among research team members, considering clinical importance, methodological quality, feasibility, and the comparability with existing RCTs and review evidence.

For pre-clinical evidence, animal studies that explored the effects of acupuncture therapies (including manual acupuncture, electro-acupuncture, acupressure, auricular acupressure, and auricular acupuncture) were included without restriction on the race, category, age, and sex of animals or study type.

### Data extraction

Two reviewers (YC and CZ) extracted the information on study design, participants’ characteristics, details of interventions and control treatments, outcome measures and results independently using a pre-designed Excel dataset. A third reviewer (SL) checked the dataset for consistency. Authors of original trials were contacted *via* emails or phone calls for clarification where important data were unclear, unavailable, or suspected as duplication. The meta-analysis in Preferred Reporting Items for Systematic Reviews and Meta-Analyses (PRISMA) guidelines were followed in selecting studies.

### Quality assessment

#### Methodological quality

Two reviewers (YC and CZ) independently assessed the methodological quality of the included studies using the Cochrane risk-of-bias tool, following the Cochrane Handbook for Systematic Reviews of Interventions (version 5.1.0). Disagreement was resolved by discussion with a third reviewer (SL) when needed. Studies were assessed as “low,” “unclear,” or “high” risk of bias for the following domains: random sequence generation, allocation concealment, blinding of participants and personnel, blinding of outcome assessors, incomplete outcome data, selective reporting, and other bias.

#### Reporting quality

Reporting quality of included trials was assessed using CONSORT and CONSORT extension for Standards for Reporting Interventions in Clinical Trials of Acupuncture (24). Detailed evaluation information is presented in Supplementary Tables 2, 3.

### Statistical analysis

Meta-analyses were performed using the Cochrane Review Manager Software (RevMan 5.3). Dichotomous data was presented with risk ratio (RR) and 95% confidence interval (CI), while continuous data was presented with mean difference (MD) and 95% CI. A random effects model was used for all meta-analyses. Statistical heterogeneity was assessed by *Chi*-square tests. Two-sided *p* < 0.05 was considered statistically significant.

Included trials were grouped based on comparisons and interventions. Subgroup analyses were planned to explore the potential sources of clinical heterogeneity. Where sufficient data were available, subgroup analyses would be conducted based on baseline number of ventricular premature beats measured by 24-h Holter (< or ≥10,000 per 24 h; < or ≥10% of total heart contractions), age (< or ≥65 years old) and treatment duration of acupuncture (< or ≥1 month). Sensitivity analyses were conducted with regards to risk of bias judgments. A funnel plot and Egger’s linear regression test were planned to detect publication bias when at least 10 studies were included in a meta-analysis. The certainty of evidence was assessed using the Grading of Recommendations Assessment, Development and Evaluation (GRADE) approach.

## Results

### Study selection

A total of 479 records were identified through database search and references of included studies and related systematic reviews (Figure 1). After removing duplicates, a total of 283 reports were identified and screened based on their titles and abstracts. A total of 44 full-text reports were retrieved and assessed for eligibility. Nine of them (2.05%) met the selection criteria and were included in this systematic review and meta-analysis. Among these nine included studies, three studies evaluated the efficacy of body acupuncture using sham control (25–27), four trials assessed the add-on effects of body acupuncture (28–31), and two trials investigated the add-on effects of auricular acupressure (32, 33) for PVCs (Table 1 and Figure 1).

**TABLE 1:** Baseline characteristics of included studies.

Article ID (References)	Setting (In./Out.)	Sample size (IG/CG)	Frequency of PVBs	Lown Grading	Period since first diagnosed with PVCs	Age range (average)	No. of male/Female	Intervention	Control	Outcomes
Zhao and Wang (31)	NS	39/39	≥500 PVBs per 24 h	≥2	NS	≤80	42/36	Body acupuncture + RC^1^	RC^1^	Effective rate,^1^ safety outcome (adverse events)
Lin et al. (26)	In. and Out.	53/53	NS	NS	N	18–70 (44.85)	36/70	Body acupuncture	Sham acupuncture	Effective rate,^1^ TCM score, safety outcome^1^
Li (25)	NS	33/33	≥500 PVBs per 24 h	1–2	1–34 months	20–60 (45.44)	35/31	Body acupuncture	Sham acupuncture	Effective rate,^1^ 24-h Holter (heart rate, SDNN, rMSSD), adverse events
Yin et al. (33)	NS	100/100	NS	NS	NS	18–30	104/96	Auricular acupressure + RC^2^	RC^2^	Effective rate^1^
Le et al. (32)	In.	40/40	NS	NS	5.2 years on average	66.85 on average	27/53	Auricular acupressure + RC^1^	RC^1^	Effective rate^1^, symptom improvement^1^, safety outcome^2^
Li et al. (34)	NS	65/65	NS	NS	4.8 years on average	18–75 (55.6)	69/61	Body acupuncture + Mexiletine	Mexiletine	Effective rate^2^, LVDD, LVESD, CD4 + cells and CD8 + cells, MACE^1^
Ma and Li (28)	Out.	30/30	≥500 PVBs per 24 h	1–2	2–7 years (3.56 years on average)	23–68 (32.15)	26/34	Body acupuncture + Amiodarone	Amiodarone	Effective rate^1^, TCM score, adverse events
Zou (27)	Out.	31/30	≥500 PVBs per 24 h	NS	1 month to 14.3 years (3.23 on average)	18–79 (53.9)	20/41	Body acupuncture	Sham acupuncture	Effective rate^1^, TCM score, 24-Holter (heart rate, heart rate variation), SF-36, adverse events
Yuan and Ai (29)	NS	35/31	NS	2–4a	0.5–13.5 years (5.4 on average)	40–65 (48.5)	35/31	Body acupuncture + Mexiletine	Mexiletine	Effective rate^1^, symptom improvement^2^

CG, control group; Effective rate^1^, number of ventricular premature beats reduced ≥ 50% measured by Holter at the end of treatment compared to baseline; Effective rate^2^, did not report how effective rate was calculated; IG, intervention group; In., inpatient department; LVDD, left ventricular end-diastolic diameter; LVESD, left ventricular end-systolic diameter; MACE^1^, death, revascularization, myocardial infarction; NS, not stated; Out., Outpatient department; rMSSD, root mean square of successive differences; RC^1^, guideline-recommended therapies (unspecified); RC^2^, Instructions on health care; Safety outcome^1^, liver function, kidney function, adverse events; Safety outcome^2^, routine testing of blood, urine and stool, liver and kidney function; SDNN, standard deviation in N-N intervals; Symptom improvement^1^, patient-reported of over 30% in symptom improvement; Symptom improvement^2^, patient-reported improvement in symptoms; TCM score, a grading system based on symptomatic performance according to traditional Chinese medicine theory; PVBs, premature ventricular beats; PVCs: premature ventricular complexes.

### Characteristics of included studies

Among all included studies, two RCTs recruited participants sorely from the outpatient department; one trial included participants only from the inpatient department; one trial recruited participants from both outpatient and inpatient department; the remaining five studies did not report information on the setting of their trials.

A total of 847 participants were included in the nine included RCTs. The sample size of each trial ranged from 60 to 200. There were more female participants than male participants (453 vs. 394). The average age of patients ranged from 32.15 to 66.85 years, with their disease duration varied greatly from 1 month to 14.3 years (Table 1). Four studies recruited patients with over 500 PVBs measured by 24-h Holter, while the remaining five trials did not specify the number of PVBs. In terms of the severity of PVCs, two RCTs recruited patients with a Lown Grading between Grade 1 and Grade 2; one trial recruited patients of Lown Grade 2 to Grade 4a; one study involved participants with ≥ Lown Grade 2; the other five studies did not specify the severity measured by Lown Grading (Table 1).

Details of the intervention and control methods are presented in Supplementary Table 4. Intervention therapies of the included studies were body acupuncture, auricular acupuncture and acupressure. Control methods used by the non-sham control trials were education and moderation of lifestyle, heart rate control medications, or anti-arrhythmic medications, such as metoprolol, mexiletine, and amiodarone. Two studies evaluated the add-on effects of auricular acupuncture in addition to guideline-recommended treatments (32, 33), four trials assessed the add-on benefits of body acupuncture (28, 29, 31, 34); three RCTs used sham control and explored the efficacy of body acupuncture for PVCs (25–27). In terms of outcome measures, all of the included studies reported effective rate, five studies reported different symptom-related scores (26–29, 32), one trial reported quality of life measured by SF-36 (27).

### Methodological quality

The judgments for risk of bias of included studies are presented in Table 2. Six studies were assessed as “low” risk of bias as they used either random number table or computer software to generate random sequence, the other three studies were of “unclear” risk of bias in this domain because relevant information could not be identified from their publications. Only three studies used envelopes for allocation concealment and therefore were assessed as “low” risk of bias in this domain. The others did not report how allocation concealment was conducted and were assessed as “unclear” risk of bias. Since the number of PVBs measured by 24-h Holter and the effective rate based on the number of PVBs are objective outcomes, we consider the influence of potential bias due to lack of appropriate blinding of outcome assessors was limited and therefore all studies were evaluated with “low risk” of bias in this domain. While the risks of performance bias were assessed as “unclear” due to unblinding of participants and personnel. All studies were assessed with “low” risk of bias in incomplete outcome data reporting as they either reported no missing data or the drop-out rates were balanced between-group and negligible for data analyses. In terms of selective reporting, one trial was assessed as “high” risk of bias because it did not report follow-up results; two studies were “low” risk of bias because they included all expected outcomes predefined in the method section though study protocols were not published. The other included trials were assessed as “unclear” risk of bias in this domain because they did not register/publish protocols or clearly list outcome measures in the methods section of the published articles. No other source of bias was detected from the included trials.

**TABLE 2:** Risk of bias assessment.

Article ID (References)	Randomization method	Allocation concealment	Blinding of participants^a^	Blinding of personnel^a^	Blinding of outcome assessment^a^	Incomplete outcome data	Selective reporting	Other bias
Zhao and Wang (31)	Low	Unclear^2^	Unclear	Unclear	Low	Low	Unclear^3^	Low
Lin et al. (26)	Low	Unclear^2^	Low	Low	Low	Low	Unclear^3^	Low
Li (25)	Low	Low	Low	Low	Low	Low	Low	Low
Yin et al. (33)	Unclear^1^	Unclear^2^	Unclear	Unclear	Low	Low	High^1^	Low
Le et al. (32)	Unclear^1^	Unclear^2^	Unclear	Unclear	Low	Low	Unclear^3^	Low
Li et al. (34)	Low	Low	Unclear	Unclear	Low	Low	Unclear^3^	Low
Ma and Li (28)	Low	Unclear^2^	Unclear	Unclear	Low	Low	Unclear^3^	Low
Zou (27)	Low	Low	Low	Low	Low	Low	Low	Low
Yuan and Ai (29)	Unclear^1^	Unclear^2^	Unclear	Unclear	Low	Low	Unclear^3^	Low

^a^The assessment of bias due to lack of blinding was based on the impact on the reduction in the number of ventricular premature beats measured with 24-h Holter; Unclear^1^, randomization method not reported; Unclear^2^, allocation concealment method not reported; Unclear^3^, trial protocol not available; High^1^, follow-up was planned but not reported.

### Effects of interventions

#### Effective rate measured by 24-h Holter

All included trials reported effective rate. These studies were grouped for meta-analyses according to comparisons.

#### Auricular acupressure + routine care vs. routine care

**FIGURE 2:**
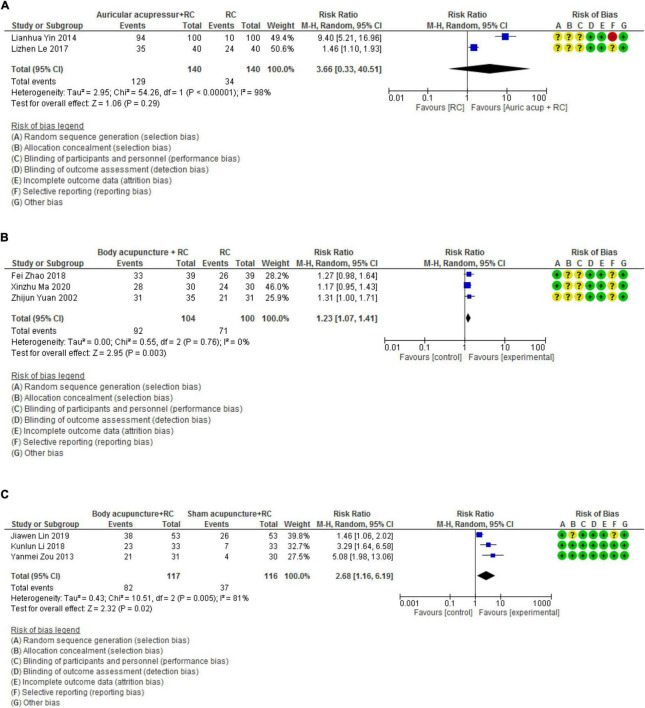
Forest plots of effective rate measured by 24-h Holter. **(A)** Forest plot_Auricular acupressure + RC vs. RC. **(B)** Forest plot_Body acupuncture + RC vs. RC. **(C)** Forest plot_Body acupuncture + RC vs. Sham acupuncture + RC. RC, routine care.

Two trials evaluated the add-on benefits of auricular acupressure for PVCs to standard therapies (including anti-arrhythmic medications and lifestyle modification instructions) (32, 33). Superior effects were observed in the combination of auricular acupressure and routine care compared to standard therapies alone in each of the included trials. However, due to high statistical heterogeneity, meta-analysis showed no significant between-group difference (RR 3.66, 95% CI [0.33, 40.51], *I*^2^ = 98%, Figure 2). Subgroup analysis and sensitivity analysis were not applicable due to insufficient number of included studies.

#### Body acupuncture + routine care vs. routine care

The results of the meta-analysis on three RCTs demonstrated that the combined effects of body acupuncture and routine care were superior to that of routine care alone (RR 1.23, 95% CI [1.07, 1.41], *I*^2^ = 0%, Figure 2) (28, 29, 31).

#### Body acupuncture vs. sham acupuncture

Three studies explored the efficacy of body acupuncture for PVCs compared with sham control (25–27). The synthesized results suggested that body acupuncture achieved higher effective rate in reducing the number of PVBs compared to sham acupuncture (RR 2.68, 95% CI [1.16, 6.19], *I*^2^ = 81%, Figure 2). We further conduct a sensitivity analysis based on the assessment of risk of bias and It was found that when the trial with “unclear” risk of bias in allocation concealment and selective reporting was excluded (26), the statistical heterogeneity (*I*^2^) was reduced to 0% and a greater superior result was detected (RR 3.83, 95% CI [2.19, 6.7], Figure 2).

### Assessment of symptom severity

#### Auricular acupressure + routine care vs. routine care

Only one study reported symptom-related effective rate, suggesting that participants using auricular acupressure and routine care achieved a higher effective rate than those using routine care alone (RR 1.44, 95% CI [1.11, 1.87]) (32). This effective rate is defined as “over 30% of patient-reported symptoms relief through baseline to end of treatment.” The measured symptoms included palpitation, chest distress, fatigue, and dizziness.

#### Body acupuncture + routine care vs. routine care

One study used accumulated symptom score to measure the change of symptoms (29). The measured symptoms included palpitation, fatigue, dizziness, and chest distress. A higher score indicated less symptom relief at the end of treatment. The result of this study showed that compared to routine care alone, body acupuncture combined with routine care produced greater symptom relief at the end of treatment (MD −1.15, 95% CI [−1.44, −0.86]). Another study evaluated palpitation score and chest-distress score separately (28). This study detected better effects of the combination of both body acupuncture and routine care compared to routine care alone in terms of palpitation relief (MD −0.15, 95% CI [−0.25, −0.05]) and chest distress relief (MD −0.16, 95% CI [−0.30, −0.02]).

#### Body acupuncture vs. sham acupuncture

One RCT reported scores measuring symptom severity showed that real acupuncture produced greater relief in all measured symptoms than sham acupuncture (27).

### Quality of life

Only one trial assessed patients’ quality of life evaluated by SF-36 (27). Results of this trial suggested that compared to sham acupuncture, body acupuncture improved patients’ quality of life with regards to physical and emotional limitations as well as general and mental health.

### Adverse events

Five studies reported 11 adverse events in intervention groups treated with acupuncture therapies and eight adverse events in control groups, most of which were local discomfort in acupuncture points (25–28, 31). Two cases of dizziness were reported with one of them treated with acupuncture (31) and the other one not reporting group allocation (25). Two cases of stomach-ache were reported in participants receiving body acupuncture (31). One case treated with body acupuncture and routine care reported chest distress (28). A total of nine cases were observed with local discomfort in the acupuncture points, including pain, subcutaneous ecchymosis, needle sticking, and local rash (26–28). All of the adverse events were reported to be mild and reversible without any additional medical management being required.

### Certainty of evidence

#### Auricular acupressure + routine care vs. routine care

The certainty of evidence on the effects of auricular acupressure adding to standard treatment compared to standard treatment alone is assessed as “low” considering substantial statistical heterogeneity and the small sample size (Table 3).

**TABLE 3:** Summary of findings.

Comparisons	Outcomes	No. of participants (studies)	Certainty of the evidence (GRADE)	Relative effect (RR, 95% CI)
Auricular acupressure + RC vs. RC	Effective rate (ventricular premature beats reduced ≥ 50%)	280 (2 RCTs)	⊕⊕○○ LOW^a,b^	3.66 (0.33, 40.51)
Body acupuncture + RC vs. RC	Effective rate (ventricular premature beats reduced ≥ 50%)	264 (4 RCTs)	⊕⊕⊕○ MODERATE^b^	1.21 (1.08, 1.36)
Body acupuncture + RC vs. Sham acupuncture + RC	Effective rate (ventricular premature beats reduced ≥ 50%)	233 (3 RCTs)	⊕⊕⊕○ MODERATE^b,c^	2.68 (1.16, 6.19)

CI, confidence interval; RC, routine care; RCTs, randomized controlled trials; RR, risk ratio. ^a^Substantial statistical heterogeneity. ^b^Small sample size limits certainty of results.

^c^Considerable statistical heterogeneity.

#### Body acupuncture + routine care vs. routine care

The certainty of evidence of the add-on effects of body acupuncture in addition to standard treatment was assessed as “moderate” because the small sample size limited the certainty of the results (Table 3).

#### Body acupuncture vs. sham acupuncture

Due to the considerable statistical heterogeneity and the small sample size of the included trials, the certainty of the evidence measuring the efficacy of body acupuncture compared to sham acupuncture was assessed as “moderate” (Table 3). In sensitivity analysis where only studies assessed with a low risk of bias in all domains were included, the certainty of evidence was “moderate” because only the small sample size limited our confidence in the final results.

### Pre-clinical evidence

Although the fundamental causes of PVCs remain largely unknown, the potential electrophysiological mechanisms include parasystole, triggered activity, mechanoelectrical feedback, enhanced automaticity, continuous activation and reflection, injury current and phase 2 re-entry, etc. (1, 4, 5).

In this review, through a systematic search of databases and title/abstract screening, sixteen studies were screened selected for full texts screening. Eleven of them were excluded because their studies were unrelated to the mechanisms of acupuncture for PVCs. Eventually five pre-clinical studies were included and were summarized in Supplementary Table 5 (35–39). Four studies explored the potential mechanisms of electro-acupuncture on body acupoints for PVCs and reported a reduction of electrical activity of sympathetic nerves in experimental animals undergoing electro-acupuncture (35, 37–39). Among the acupoints used by these four studies, Neiguan (coded as PC6, located on the anterior aspect of the forearm, between the tendons of the palmaris longus and the flexor carpi radialis, 2 B-cun proximal to the palmar wrist crease) (40) was the most frequently reported (Supplementary Table 5). Another study explored the possible underlying effects of auricular acupuncture with electrical stimulation and reported that acupuncture might have an impact on rostral venteral lateral medulla cardiovascular center (Supplementary Table 5; 36). The application of acupuncture therapies are complex whose effectiveness is potentially associated with combined effects of both the intervention stimulation and point-specific effects. Therefore, further studies investigating the mechanisms are needed to unveil the underlying pathways.

## Discussion

### General interpretation

Meta-analyses results suggested that the combination of acupuncture therapies with standard treatment was associated with a greater reduction in the frequency of PVBs compared to standard treatment alone. When compared to sham acupuncture, body acupuncture was more effective for reducing the number of PVBs. However, the certainty of the above results was limited by the small sample size of the included trials. As for the changes in patient-reported symptoms, improvement was observed by individual studies even although meta-analysis was not conducted due to variation in the components of the reported scores. It is worth noting that to alleviate symptom and improve quality of life are the key reasons for seeking clinical management for PVCs patients without ischemic or structural heart diseases. To enable comparisons among studies, validated and reliable outcome measurement instruments for symptom changes and quality of life are needed in future studies of PVCs. Moreover, acupuncture therapies were well-tolerated with mild and reversible adverse events.

### Similarities and differences with other reviews

We identified three previous systematic reviews evaluating the effects of acupuncture therapies for cardiac arrhythmia (21–23). All these three systematic reviews evaluated the treatment effects of acupuncture on various types of cardiac arrhythmias (21–23). One meta-analysis involving three trials showed that acupuncture was as effective as conventional drug for PVBs (22), another meta-analysis on five trials showed that the combination of acupuncture plus oral anti-arrhythmic drug was more effective than anti-arrhythmic drug alone (23). All these meta-analyses were conducted based on “response rate”; however, the definition of “response rate” was not consistent. While our systematic review focused on PVCs without ischemic or structural heart diseases using rigorously defined outcome measures to provide robust evidence on the effectiveness and efficacy of acupuncture therapies.

We also identified an ongoing systematic review which is designed to evaluate the effectiveness of acupuncture for symptom alleviation for PVCs with and without ischemic or structural heart diseases (41). According to the published protocol, this review includes complex acupuncture interventions therefore it will be difficult to interpret its results to identify the treatment effects of each acupuncture methods (41). In addition, the outcome measures used in the ongoing review are not precisely defined (41).

### Limitations of evidence

#### Limitations in methodological quality

Proper randomization methods and blinding of participants were insufficiently applied by the studies included in this review, which may cause potential risks in selection bias and performance bias, particularly for subjective outcomes such as symptomatic scores and quality of life (42). In addition, the lack of registered/published protocols downgraded our confidence on the design and results of the included studies (Table 2).

#### Limitations in clinical heterogeneity

Considerable heterogeneity was detected in two meta-analyses (Table 3), lowering our confidence in the overall evidence. It is worth noting that the high heterogeneity was reduced by sensitivity analysis of excluding the trial with “unclear” risk of bias in allocation concealment and selective reporting (Figure 2), emphasizing the importance of rigorous study design. Besides, baseline variations in patient characteristics and therapeutic protocols may contribute to the clinical heterogeneity of the meta-analyses (Table 1). Subgroup analysis was planned but not applicable due to insufficient number of included trials.

#### Small sample size

Small sample size of the included trials limited our confidence in the results of the review. Moreover, due to the small number of studies included in meta-analyses (n < 10), publication bias could not be evaluated.

### Implication for research

Firstly, rigorously designed studies are needed to further investigate the association between PVCs patients with variant characteristics and specific acupuncture treatment protocols, since the meta-analyses demonstrated positive results over control but with high statistical and clinical heterogeneity. Secondly, although there is controversy over the effects and blinding credibility of sham acupuncture (43–45), more sham-controlled studies with sufficient number of participants are still encouraged as they can reduce bias in evaluating subjective outcomes and evaluation of blinding credibility are needed (46). Thirdly, efforts to reduce the impact of potential bias with non-blinded studies are essential as pragmatic trials with open-labeled design have advantages in practical feasibility as well as increased extrapolation in real-world situation (47), particularly for interventions with complex, flexible, and individualized treatment protocols (48, 49). Fourthly, comprehensive outcomes should be developed and evaluated in future studies assessing not only the number of PVBs, but also patient-reported symptom severity and quality of life, because symptom control plays an important role in PVCs management considering PVCs is a relatively benign condition (50).

### Implications for clinical practice

Based on current evidence, acupuncture therapies could be used as a complementary treatment for PVCs with regards to reducing the number of premature beats, particularly in combination of routine care. There has not been consensus in specific parameters of acupuncture therapies recommended for PVCs, but practical clues can be driven from the generally applied acupuncture treatment protocols among included studies as summarized in Supplementary Table 4. For instance, as for acupuncture point selection, PC6 is the most frequently used point followed by HT7, DU10, DU11, and ST36. Furthermore, acupuncture retention duration usually ranges from 15 to 30 min and a total acupuncture therapy period is generally reported around 4 weeks.

## Conclusion

This review suggests that acupuncture could be a therapeutic option to reduce the burden of PVBs in patients without ischemic or structural heart diseases. More placebo-controlled studies with sufficient sample size, using validated and reliable outcome measures are needed to improve our certainty of the evidence and explore the potential benefit for those intolerant, fail for or decline mainstream medical treatments. More bench studies to unveil the mechanisms of acupuncture stimulation and point-specific effects for PVCs are needed.

## Data availability statement

The original contributions presented in this study are included in this article/Supplementary material, further inquiries can be directed to the corresponding author.

## Author contributions

YC, BT, and WC contributed to the conception and design of the review. YC and CZ conducted the data analysis and drafted the manuscript. SL, LZ, BT, and WC critically revised the manuscript. All authors gave final approval and agreed to be accountable for all aspects of work ensuring integrity and accuracy.

## Footnotes

1
www.chictr.org.cn/enIndex.aspx

2
https://www.anzctr.org.au/

3
https://cris.nih.go.kr/cris/index/index.do

4
https://www.clinicaltrialsregister.eu/ctr-search/search

5
https://rctportal.niph.go.jp/en/

6
https://clinicaltrials.gov

## Supplementary material

Supplementary Table 1 Search strategies.

Supplementary Table 2 Reporting quality (CONSORT).

Supplementary Table 3 Reporting quality (STRICTA).

Supplementary Table 4 Details of interventions and control measures.

Supplementary Table 5 Summary of related experimental studies.
