# Supplementary material for: Acupuncture for premature ventricular complexes without ischemic or structural heart diseases: A systematic review and meta-analysis of clinical and pre-clinical evidence
Source: Front Med (Lausanne). 2022 Dec 8;9:1019051. doi: 10.3389/fmed.2022.1019051 (PMC9773094; doi:10.3389/fmed.2022.1019051)
Supplement: Supplementary Table 1 — Search strategies. [file Table_1.DOCX]

Supplementary Material

# Supplementary Table 1 Search Strategies

| Database^a^ | Search strategy |
| --- | --- |
| PubMed | ("acupuncture"[Title/Abstract] OR "meridians"[Title/Abstract] OR "auriculotherapy"[Title/Abstract] OR "plum blossom"[Title/Abstract] OR "acupressure"[Title/Abstract] OR "ear acupuncture"[Title/Abstract] OR "ear acupressure"[Title/Abstract] OR "acupuncture therapy"[Title/Abstract] OR "laser acupuncture"[Title/Abstract] OR "seven star needle"[Title/Abstract] OR "acupuncture points"[Title/Abstract] OR "electro-acupuncture"[Title/Abstract] OR "electroacupuncture"[Title/Abstract] OR "electro acupuncture"[Title/Abstract] OR "electro-stimulation"[Title/Abstract] OR "electro-stimulation"[Title/Abstract]) AND ("ventricular premature beat"[Title/Abstract] OR "premature ventricular beat"[Title/Abstract] OR "ventricular premature contraction"[Title/Abstract] OR "premature ventricular contraction"[Title/Abstract] OR "ventricular premature complex"[Title/Abstract] OR "premature ventricular complex"[Title/Abstract] OR "ventricular ectopic beat"[Title/Abstract] OR "ectopic ventricular beat"[Title/Abstract] OR "ventricular extrasystole"[Title/Abstract] OR "ventricular premature beats"[Title/Abstract] OR "premature ventricular beats"[Title/Abstract] OR "ventricular premature contractions"[Title/Abstract] OR "premature ventricular contractions"[Title/Abstract] OR "ventricular premature complexes"[Title/Abstract] OR "premature ventricular complexes"[Title/Abstract] OR "ventricular ectopic beats"[Title/Abstract] OR "ectopic ventricular beats"[Title/Abstract] OR "ventricular extrasystoles"[Title/Abstract]) |
| Chinese Biomedical Database (Chinese language database) | (("埋藏疗法"[常用字段:智能] OR "埋线"[常用字段:智能]) OR ("电磁针"[常用字段:智能] OR "梅花针"[常用字段:智能] OR "穴位按压"[常用字段:智能] OR "穴位按摩"[常用字段:智能] OR "指压穴位"[常用字段:智能] OR "耳压"[常用字段:智能] OR "耳针"[常用字段:智能] OR "耳穴"[常用字段:智能] OR "耳豆"[常用字段:智能]) OR ("针刺"[常用字段:智能] OR "针灸"[常用字段:智能] OR "针法"[常用字段:智能] OR "刺法"[常用字段:智能] OR "体针"[常用字段:智能] OR "腹针"[常用字段:智能] OR "温针"[常用字段:智能] OR "火针"[常用字段:智能] OR "电针"[常用字段:智能])) AND ("期前收缩"[常用字段:智能] OR "期外收缩"[常用字段:智能] OR "室性期前收缩"[常用字段:智能] OR "室性期前收缩"[常用字段:智能] OR "早搏"[常用字段:智能] OR "室性早搏"[常用字段:智能] OR "室早"[常用字段:智能]) |

Notes: ^a^ The four English-language databases were PubMed, EMBASE, Cochrane Library and Cumulative Index to Nursing and Allied Health Literature. The four Chinese-language databases included were Chinese Biomedical Database (SinoMed), Chinese National Knowledge Infrastructure (CNKI), Chongqing VIP Database (VIP) and Wanfang Database (Wanfang). The online clinical trial registration websites were the Chinese Clinical Trial Register (www.chictr.org.cn/enIndex.aspx), Australian New Zealand Clinical Trials Registry (ANZCTR, https://www.anzctr.org.au/), Clinical Research Information Service (CRiS), Republic of Korea (https://cris.nih.go.kr/cris/index/index.do), EU Clinical Trials Register (EU-CTR, https://www.clinicaltrialsregister.eu/ctr-search/search) and the Japan Primary Registries Network (JPRN, https://rctportal.niph.go.jp/en/) and ClinicalTrial.gov (https://clinicaltrials.gov).
