# Supplementary material for: Acupuncture for premature ventricular complexes without ischemic or structural heart diseases: A systematic review and meta-analysis of clinical and pre-clinical evidence
Source: Front Med (Lausanne). 2022 Dec 8;9:1019051. doi: 10.3389/fmed.2022.1019051 (PMC9773094; doi:10.3389/fmed.2022.1019051)
Supplement: Supplementary Table 2 — Reporting quality (CONSORT). [file Table_2.DOCX]

Supplementary Material

# Supplementary Table 2 Reporting Quality (CONSORT)

| Section | Topic | Item | Study | | | | | | | | |
| --- | --- | --- | --- | --- | --- | --- | --- | --- | --- | --- | --- |
|  |  |  | Fei Zhao 2018 (1) | Jiawen Lin 2019 (2) | Kunlun Li 2018 (3) | Lianhua Yin 2014 (4) | Lizhen Le 2017 (5) | Min Li 2017 (6) | Xinzhu Ma 2020 (7) | Yanmei Zou 2013 (8) | Zhijun Yuan 2002 (9) |
| Title and abstract | | 1a | No | No | No | No | No | No | No | Yes | No |
|  |  | 1b | Yes | Yes | Yes | Yes | Yes | Yes | Yes | Yes | No |
| Introduction | Background and objectives | 2a | No | Yes | Yes | No | Yes | Yes |  | Yes | No |
|  |  | 2b | No | Yes | Yes | No | Yes | Yes | Yes | Yes | Yes |
| Methods | Trial design | 3a | Yes | Yes | Yes | Yes | Yes | Yes | Yes | Yes | Yes |
|  |  | 3b | Yes | Yes | Yes | Yes | Yes | Yes | Yes | Yes | Yes |
|  | Participants | 4a | Yes | Yes | Yes | Yes | Yes | Yes | Yes | Yes | Yes |
|  |  | 4b | No | Yes | Yes | No | Yes | No | Yes | Yes | No |
|  | Interventions | 5 | Yes | Yes | Yes | Yes | Yes | Yes | Yes | Yes | Yes |
|  | Outcomes | 6a | Yes | Yes | Yes | Yes | Yes | Yes | Yes | Yes | Yes |
|  |  | 6b | N/A | N/A | N/A | N/A | N/A | N/A | N/A | N/A | N/A |
|  | Sample size | 7a | No | No | No | No | No | No | No | No | No |
|  |  | 7b | N/A | N/A | N/A | N/A | N/A | N/A | N/A | N/A | N/A |
|  | Randomization |  | Yes | Yes | Yes | Yes | Yes | Yes | Yes | Yes | Yes |
|  | Sequence generation | 8a | No | Yes | Yes | No | No | No | Yes | Yes | No |
|  |  | 8b | Yes | Yes | Yes | Yes | Yes | Yes | Yes | Yes | Yes |
|  | Allocation concealment | 9 | No | No | Yes | No | No | No | No | Yes | No |
|  | Implementation | 10 | No | No | No | No | No | No | No | Yes | No |
|  | Blinding | 11a | N/A | Yes | Yes | N/A | N/A | N/A | N/A | N/A | N/A |
|  |  | 11b | N/A | Yes | Yes | N/A | N/A | N/A | N/A | N/A | N/A |
|  | Statistical methods | 12a | Yes | Yes | Yes | Yes | Yes | Yes | Yes | Yes | Yes |
|  |  | 12b | N/A | N/A | N/A | N/A | N/A | N/A | N/A | N/A | N/A |
| Results | Participant flow | 13a | No | Yes | No | Yes | Yes | Yes | Yes | Yes | Yes |
|  |  | 13b | No | N/A | N/A | N/A | N/A | N/A | N/A | Yes | N/A |
|  | Implementation of intervention |  | Yes | Yes | Yes | Yes | Yes | Yes | Yes | Yes | Yes |
|  | Recruitment | 14a | No | No | Yes | Yes | Yes | Yes | No | Yes | Yes |
|  |  | 14b | N/A | N/A | N/A | N/A | N/A | N/A | N/A | N/A | N/A |
|  | Baseline data | 15 | No | No | Yes | No | Yes | No | No | Yes | No |
|  | Numbers analyzed | 16 | Yes | Yes | Yes | Yes | Yes | Yes | Yes | Yes | Yes |
|  | Outcomes and estimation | 17a | Yes | Yes | Yes | No | Yes | Yes | Yes | Yes | Yes |
|  |  | 17b | No | Yes | No | No | No | No | No | No | No |
|  | Ancillary analyses | 18 | N/A | N/A | N/A | N/A | N/A | N/A | N/A | N/A | N/A |
|  | Harms | 19 | Yes | Yes | Yes | Yes | Yes | Yes | Yes | Yes | Yes |
| Discussion | Limitations | 20 | No | No | No | No | No | No | No | Yes | No |
|  | Generalizability | 21 | No | No | No | No | No | No | No | No | No |
|  | Interpretation | 22 | No | No | No | No | No | No | No | Yes | No |
| Other Information | Registration | 23 | No | No | No | No | No | No | No | No | No |
|  | Protocol | 24 | No | No | Yes | No | No | No | No | Yes | No |
|  | Funding | 25 | No | Yes | No | No | No | No | No | No | No |

Notes: CONSORT: Consolidated Standards of Reporting Trials; N/A: not applicable; details of item contents are presented below (these two checklists were cited from: Boutron I, Altman DG, Moher D, Schulz KF, Ravaud P, DJ C, et al. CONSORT Statement for Randomized Trials of Nonpharmacologic Treatments: A 2017 Update and a CONSORT Extension for Nonpharmacologic Trial Abstracts. Annals of Internal Medicine. American College of Physicians; 2017 Jul 4;167(1):40. PMID: 28630973).

| **Item** | **CONSORT 2010 Statement Checklist item** |
| --- | --- |
| 1.a | Identification as a randomized trial in the title |
| 1.b | Structured summary of trial design, methods, results, and conclusions |
| 2.a | Scientific background and explanation of rationale |
| 2.b | Specific objectives or hypotheses |
| 3.a | Description of trial design (e.g., parallel, factorial) including allocation ratio |
| 3.b | Important changes to methods after trial commencement (e.g. eligibility criteria), with reasons |
| 4.a | Eligibility criteria for participants |
| 4.b | Settings and locations where the data were collected |
| 5 | The interventions for each group with sufficient details to allow replication, including how and when they were actually administered |
| 6.a | Completely defined pre-specified primary and secondary outcome measures, including how and when they were assessed |
| 6.b | Any changes to trial outcomes after the trial commenced with reasons |
| 7.a | How sample size was determined |
| 7.b | When applicable, explanation of any interim analyses and stopping guidelines |
| 8.a | Method used to generate the random allocation sequence |
| 8.b | Type of randomization; details of any restriction (e.g., blocking and block size) |
| 9 | Mechanism used to implement the random allocation sequence (e.g., sequentially numbered containers), describing any steps taken to conceal the sequence until interventions were assigned |
| 10 | Who generated the random allocation sequence, who enrolled participants, and who assigned participants to interventions |
| 11.a | If done, who was blinded after assignment to interventions (e.g. participants, care providers, those assessing outcomes) and how |
| 11.b | If relevant, description of the similarity of interventions |
| 12.a | Statistical methods used to compare groups for primary and secondary outcomes |
| 12.b | Methods for additional analyses, such as subgroup analyses and adjusted analyses |
| 13.a | For each group, the numbers of participants who were randomly assigned, received intended treatment, and were analyzed for the primary outcome |
| 13.b | For each group, losses and exclusions after randomization, together with reasons |
| 14.a | Dates defining the periods of recruitment and follow-up |
| 14.b | Why the trial ended or was stopped |
| 15 | A table showing baseline demographic and clinical characteristics for each group |
| 16 | For each group, number of participants (denominator) included in each analysis and whether the analysis was by original assigned groups |
| 17.a | For each primary and secondary outcome, results for each group, and the estimated effect size and its precision (e.g., 95% confidence interval) |
| 17.b | For binary outcomes, presentation of both absolute and relative effect sizes is recommended |
| 18 | Results of any other analyses performed, including subgroup analyses and adjusted analyses, distinguishing pre-specified from exploratory |
| 19 | All important harms or unintended effects in each group |
| 20 | Trial limitations, addressing sources of potential bias, imprecision, and, if relevant, multiplicity of analyses |
| 21 | Generalizability (external validity, applicability) of the trial findings |
| 22 | Interpretation consistent with results, balancing benefits and harms, and considering other relevant evidence |
| 23 | Registration number and name of trial registry |
| 24 | Where the full trial protocol can be accessed, if available |
| 25 | Sources of funding and other support (e.g., supply of drugs); role of funders |

**References**

1. Zhao F, Wang N. [Clinical Study of 78 Cases Diagnosed with Frequent Premature Ventricular Complexes Treated with Acupuncture] (Article in Chinese Medicine). *Guide of China Medicine* (2018) 16(9):185. Epub 20181230. doi: 10.15912/j.cnki.gocm.2018.09.157.

2. Lin J, Chen J, Shen R, Zeng H, Zhang X, Lu W, et al. [Observation on Treating Functional Ventricular Premature Beats with Somatic Symptom Disorders by Acupuncture] (Article in Chinese). *Clinical Journal of Chinese Medicine* (2019) 11(35):95-8. Epub 20200804. doi: 10.3969/j.issn.1674-7860.2019.35.036.

3. Li K. [Immediate Effect of Acupuncture at Qu Ze on Heart Rate Variability in Patients with Functional Ventricular] (Article in Chinese Language) [Masters]: Guangzhou University of Chinese Medicine (2018).

4. Yin L, Xu Y, Huang S. [the Treatment of Auricular-Plaster Therapy on 100 Patients with Ventricular Premature Beat] (Article in Chinese Language). Asia-Pacific Traditional Medicine (2014) 10(23):43-4. Epub 20151026.

5. Le L, Yan J, Li Y, Xiao G, Zeng K. [Auricular Acupressure for Ventricular Premature Complexes in the Absence of Structural Heart Diseases Involving 40 Cases] (Article in Chinese Language). *Yunnan Journal of Traditional Chinese Medicine and Materia Medica* (2017) 38(3):105-6. Epub 20170921. doi: 10.16254/j.cnki.53-1120/r.2017.03.051.

6. Li M, Wang P, Xu M. [Efficiency and Effects of Immune Function of Electroacupuncture at Lingtai and Shendao Acupoints on the Premature Beats Patients] (Article in Chinese Language). *China Journal of Traditional Chinese Medicine and Pharmacy* (2017) (6):2821-4. Epub 20191110.

7. Ma X, Li C. [Clinical Observation on 30 Cases of Ventricular Premature Beat Treated by Western Medicine Combined with Press-Needle] (Article in Chinese Language). *Chinese Journal of Ethnomedicine and Ethnopharmacy* (2020) 29(11):92-4. Epub 20210311.

8. Zou Y. [the Randomized Controlled Study of Abdominal Acupuncture Treatment for Functional Premature Ventricular Contractions] (Article in Chinese Language) [Doctor]: Guangzhou University of Chinese Medicine (2013).

9. Yuan Z, Ai B. [Clinical Study of Acupuncture Combined with Medications for Premature Ventricular Complexes] (Article in Chinese Medicine). *Zhongguo Zhong Xi Yi Jie He Za Zhi* (2002) 22(4):312-3. Epub 20021231.
