# Supplementary material for: Acupuncture for premature ventricular complexes without ischemic or structural heart diseases: A systematic review and meta-analysis of clinical and pre-clinical evidence
Source: Front Med (Lausanne). 2022 Dec 8;9:1019051. doi: 10.3389/fmed.2022.1019051 (PMC9773094; doi:10.3389/fmed.2022.1019051)
Supplement: Supplementary Table 3 — Reporting quality (STRICTA). [file Table_3.DOCX]

Supplementary Material

# Supplementary Table 3 Reporting Quality (STRICTA)

| Item | Detail | Study | | | | | | | | |
| --- | --- | --- | --- | --- | --- | --- | --- | --- | --- | --- |
|  |  | Fei Zhao 2018 (1) | Jiawen Lin 2019 (2) | Kunlun Li 2018 (3) | Lianhua Yin 2014 (4) | Lizhen Le 2017 (5) | Min Li 2017 (6) | Xinzhu Ma 2020 (7) | Yanmei Zou 2013 (8) | Zhijun Yuan 2002 (9) |
| 1. Acupuncture rationale | 1a) Style of acupuncture (e.g. Traditional Chinese Medicine, Japanese, Korean, Western medical, Five Element, ear acupuncture, etc) | Yes | Yes | Yes | Yes | Yes | Yes | Yes | Yes | Yes |
|  | 1b) Reasoning for treatment provided, based on historical context, literature sources, and/or consensus methods, with references where appropriate | No | No | Yes | No | No | Yes | Yes | Yes | No |
|  | 1c) Extent to which treatment was varied | Yes | Yes | Yes | Yes | Yes | Yes | Yes | Yes | Yes |
| 2. Details of needling | 2a) Number of needle insertions per subject per session (mean and range where relevant) | Yes | Yes | Yes | Yes | Yes | Yes | Yes | Yes | Yes |
|  | 2b) Names (or location if no standard name) of points used (uni/bilateral) | Yes | Yes | Yes | Yes | Yes | Yes | Yes | Yes | Yes |
|  | 2c) Depth of insertion, based on a specified unit of measurement, or on a particular tissue level | Yes | No | Yes | N/A | N/A | No | N/A | Yes | No |
|  | 2d) Response sought (e.g. *de qi* or muscle twitch response) | Yes | No | Yes | Yes | Yes | No | Yes | Yes | No |
|  | 2e) Needle stimulation (e.g. manual, electrical) | Yes | Yes | Yes | Yes | Yes | Yes | Yes | Yes | Yes |
|  | 2f) Needle retention time | No | Yes | Yes | Yes | Yes | Yes | Yes | Yes | Yes |
| 3. Treatment regimen | 3a) Number of treatment sessions | Yes | Yes | Yes | Yes | Yes | Yes | Yes | Yes | Yes |
|  | 3b) Frequency and duration of treatment sessions | Yes | Yes | Yes | Yes | Yes | Yes | Yes | Yes | Yes |
| 4. Other components of treatment | 4a) Details of other interventions administered to the acupuncture group (e.g. moxibustion, cupping, herbs, exercises, lifestyle advice) | Yes | Yes | Yes | Yes | Yes | Yes | Yes | Yes | Yes |
|  | 4b) Setting and context of treatment, including instructions to practitioners, and information and explanations to patients | No | No | No | No | Yes | No | No | Yes | No |
| 5. Practitioner background | 5) Description of participating acupuncturists (qualification or professional affiliation, years in acupuncture practice, other relevant experience) | No | No | No | No | No | No | No | No | No |
| 6. Control or comparator interventions | 6a) Rationale for the control or comparator in the context of the research question, with sources that justify this choice | Yes | Yes | Yes | No | No | No | No | Yes | No |
|  | 6b) Precise description of the control or comparator. If sham acupuncture or any other type of acupuncture-like control is used, provide details as for Items 1 to 3 above. | No | Yes | Yes | Yes | No | Yes | Yes | Yes | Yes |

Notes: N/A: not applicable; "No": the correspondent item was insufficiently reported; "Yes": the correspondent item was adequately reported; the above checklist was cited from: MacPherson H, Altman DG, Hammerschlag R, Youping L, Taixiang W, White A, Moher D; STRICTA Revision Group. Revised STandards for Reporting Interventions in Clinical Trials of Acupuncture (STRICTA): extending the CONSORT statement. PLoS Med. 2010 Jun 8;7(6):e1000261. PMID: 2054399.

**References**

1. Zhao F, Wang N. [Clinical Study of 78 Cases Diagnosed with Frequent Premature Ventricular Complexes Treated with Acupuncture] (Article in Chinese Medicine). *Guide of China Medicine* (2018) 16(9):185. Epub 20181230. doi: 10.15912/j.cnki.gocm.2018.09.157.

2. Lin J, Chen J, Shen R, Zeng H, Zhang X, Lu W, et al. [Observation on Treating Functional Ventricular Premature Beats with Somatic Symptom Disorders by Acupuncture] (Article in Chinese). *Clinical Journal of Chinese Medicine* (2019) 11(35):95-8. Epub 20200804. doi: 10.3969/j.issn.1674-7860.2019.35.036.

3. Li K. [Immediate Effect of Acupuncture at Qu Ze on Heart Rate Variability in Patients with Functional Ventricular] (Article in Chinese Language) [Masters]: Guangzhou University of Chinese Medicine (2018).

4. Yin L, Xu Y, Huang S. [the Treatment of Auricular-Plaster Therapy on 100 Patients with Ventricular Premature Beat] (Article in Chinese Language). Asia-Pacific Traditional Medicine (2014) 10(23):43-4. Epub 20151026.

5. Le L, Yan J, Li Y, Xiao G, Zeng K. [Auricular Acupressure for Ventricular Premature Complexes in the Absence of Structural Heart Diseases Involving 40 Cases] (Article in Chinese Language). *Yunnan Journal of Traditional Chinese Medicine and Materia Medica* (2017) 38(3):105-6. Epub 20170921. doi: 10.16254/j.cnki.53-1120/r.2017.03.051.

6. Li M, Wang P, Xu M. [Efficiency and Effects of Immune Function of Electroacupuncture at Lingtai and Shendao Acupoints on the Premature Beats Patients] (Article in Chinese Language). *China Journal of Traditional Chinese Medicine and Pharmacy* (2017) (6):2821-4. Epub 20191110.

7. Ma X, Li C. [Clinical Observation on 30 Cases of Ventricular Premature Beat Treated by Western Medicine Combined with Press-Needle] (Article in Chinese Language). *Chinese Journal of Ethnomedicine and Ethnopharmacy* (2020) 29(11):92-4. Epub 20210311.

8. Zou Y. [the Randomized Controlled Study of Abdominal Acupuncture Treatment for Functional Premature Ventricular Contractions] (Article in Chinese Language) [Doctor]: Guangzhou University of Chinese Medicine (2013).

9. Yuan Z, Ai B. [Clinical Study of Acupuncture Combined with Medications for Premature Ventricular Complexes] (Article in Chinese Medicine). *Zhongguo Zhong Xi Yi Jie He Za Zhi* (2002) 22(4):312-3. Epub 20021231.
