# Supplementary material for: Acupuncture for premature ventricular complexes without ischemic or structural heart diseases: A systematic review and meta-analysis of clinical and pre-clinical evidence
Source: Front Med (Lausanne). 2022 Dec 8;9:1019051. doi: 10.3389/fmed.2022.1019051 (PMC9773094; doi:10.3389/fmed.2022.1019051)
Supplement: Supplementary Table 4 — Details of interventions and control measures. [file Table_4.DOCX]

Supplementary Material

# Supplementary Table 4 Details of Interventions and Control Measures

| Article ID (first author, year) | Intervention | Acupoints ^a^ | Duration of manipulation | Duration of retention | Acupuncture treatment frequency | Acupuncture therapy period | Control |
| --- | --- | --- | --- | --- | --- | --- | --- |
| Fei Zhao 2018 (1) | Body acupuncture + routine care | DU10, DU11 | 2 to 3 minutes | Not reported | Once per day | 4 weeks | RC^1^ |
| Jiawen Lin 2019 (2) | Body acupuncture | HT7、PC6、HT5、BL15、RN14、BL14、RN17、LR3、DU24、DU20、ST36、SP6 | 1 minute | 30 minutes | 3 times per week | 12 weeks | Sham acupuncture |
| Kunlun Li 2018 (3) | Body acupuncture | PC3 | 5 minutes | 30 minutes | Once | 30 minutes | Sham acupuncture |
| Lianhua Yin 2014 (4) | Auricular acupressure + RC^2^ | Auricular a acupressure | 3 to 5 minutes per time | Not applicable | 1 to 3 times per day | Not reported | RC^2^ |
| Lizhen Le 2017 (5) | Auricular acupressure + RC^1^ | Auricular a acupressure | not reported | Not applicable | 6 times per day | 2 weeks | RC^1^ |
| Min Li 2017 (6) | Body acupuncture + Mexiletine | DU10, DU11 | not reported | 20 minutes | Once per day | 20 days | Mexiletine |
| Xinzhu Ma 2020 (7) | Body acupuncture + Amiodarone | PC6 | 20 presses per time | 3 to 5 days | 5 times per day | 1 month | Amiodarone |
| Yanmei Zou 2013 (8) | Body acupuncture | RN12、RN10、RN6、RN4 | not reported | 30 minutes | 3 times per week | 4 weeks | Sham acupuncture |
| Zhijun Yuan 2002 (9) | Body acupuncture + Mexiletine | PC6、HT7、EX-B2、ST36 | not reported | 30 minutes | Once per day | 1 month | Mexiletine |

Notes: RC^1^, routine care with guideline recommended therapies; RC^2^, routine care with instructions on health care; ^a^, acupuncture points were coded according to “Lim S. WHO Standard Acupuncture Point Locations. Evid Based Complement Alternat Med. 2010 Jun;7(2):167-8. doi: 10.1093/ecam/nep006. Epub 2009 Feb 24. PMID: 19204011; PMCID: PMC2862941.”

**References**

1. Zhao F, Wang N. [Clinical Study of 78 Cases Diagnosed with Frequent Premature Ventricular Complexes Treated with Acupuncture] (Article in Chinese Medicine). *Guide of China Medicine* (2018) 16(9):185. Epub 20181230. doi: 10.15912/j.cnki.gocm.2018.09.157.

2. Lin J, Chen J, Shen R, Zeng H, Zhang X, Lu W, et al. [Observation on Treating Functional Ventricular Premature Beats with Somatic Symptom Disorders by Acupuncture] (Article in Chinese). *Clinical Journal of Chinese Medicine* (2019) 11(35):95-8. Epub 20200804. doi: 10.3969/j.issn.1674-7860.2019.35.036.

3. Li K. [Immediate Effect of Acupuncture at Qu Ze on Heart Rate Variability in Patients with Functional Ventricular] (Article in Chinese Language) [Masters]: Guangzhou University of Chinese Medicine (2018).

4. Yin L, Xu Y, Huang S. [the Treatment of Auricular-Plaster Therapy on 100 Patients with Ventricular Premature Beat] (Article in Chinese Language). Asia-Pacific Traditional Medicine (2014) 10(23):43-4. Epub 20151026.

5. Le L, Yan J, Li Y, Xiao G, Zeng K. [Auricular Acupressure for Ventricular Premature Complexes in the Absence of Structural Heart Diseases Involving 40 Cases] (Article in Chinese Language). *Yunnan Journal of Traditional Chinese Medicine and Materia Medica* (2017) 38(3):105-6. Epub 20170921. doi: 10.16254/j.cnki.53-1120/r.2017.03.051.

6. Li M, Wang P, Xu M. [Efficiency and Effects of Immune Function of Electroacupuncture at Lingtai and Shendao Acupoints on the Premature Beats Patients] (Article in Chinese Language). *China Journal of Traditional Chinese Medicine and Pharmacy* (2017) (6):2821-4. Epub 20191110.

7. Ma X, Li C. [Clinical Observation on 30 Cases of Ventricular Premature Beat Treated by Western Medicine Combined with Press-Needle] (Article in Chinese Language). *Chinese Journal of Ethnomedicine and Ethnopharmacy* (2020) 29(11):92-4. Epub 20210311.

8. Zou Y. [the Randomized Controlled Study of Abdominal Acupuncture Treatment for Functional Premature Ventricular Contractions] (Article in Chinese Language) [Doctor]: Guangzhou University of Chinese Medicine (2013).

9. Yuan Z, Ai B. [Clinical Study of Acupuncture Combined with Medications for Premature Ventricular Complexes] (Article in Chinese Medicine). *Zhongguo Zhong Xi Yi Jie He Za Zhi* (2002) 22(4):312-3. Epub 20021231.
