# Supplementary material for: Acupuncture for premature ventricular complexes without ischemic or structural heart diseases: A systematic review and meta-analysis of clinical and pre-clinical evidence
Source: Front Med (Lausanne). 2022 Dec 8;9:1019051. doi: 10.3389/fmed.2022.1019051 (PMC9773094; doi:10.3389/fmed.2022.1019051)
Supplement: Supplementary Table 5 — Summary of related experimental studies. [file Table_5.DOCX]

Supplementary Material

# Supplementary Table 5 Summary of Related Experimental Studies

| Study | Acupuncture points^1^ | Stimulation | Effects on ventricular premature complexes | Potential mechanisms | Animal models |
| --- | --- | --- | --- | --- | --- |
| Peng Li 2013 (1) | Median nerve underneath acupoint PC5 or deep peroneal nerve underneath ST36 | A low-current and low-frequency stimulation | Inhibitory effect on ventricular extrasystoles | Activate arcuate nucleuseventral periaqueductal gray enuclei raphe pathway and release endorphin, enkephaline, gamma-aminobutyric acid, 5-hydroxytryptamine, etc., thus inhibiting the rostral ventrolateral medulla, decreasing sympathetic outflow | Rabbits |
| Elizabeth R Magden 2016 (2) | PC6 and HT7 | Acupuncture needles with Low-level laser therapy (2 minutes per site bilaterally) | Decrease the mean number of ventricular premature beats per minute | 1) Increase the levels of α-endorphins and dynorphin in the periaqueductal gray matter of the brain, which in turn decreases the release of norepinephrine and dopamine, subsequently reducing the sympathetic stimulation in the heart; 2) Pathways of reduced sympathetic stimulation | Chimpanzees |
| Yanjun Duan 2009 (3) | PC6, LU7, LU9, PC7, ST36, ST34, GB34, GB31 | Electro-acupuncture | Decrease the number of ventricular premature beats while increase heart rate | Increase the electrical activity of vagus nerves and reduce the electrical activity of sympathetic nerves | Rats |
| Hui Bian 2005 (4) | Thoracopneumatic Area (auricular acupuncture) | Electro-acupuncture | Shorten the length of period with ventricular premature beats | Acupuncture might have an impact on rostral venteral lateral medulla cardiovascular center | Rabbits |
| Hui Zhong 2002 (5) | PC6 and HT7 | Electro-acupuncture | Reduce the number of ventricular premature beats | Specific mechanisms are to be further explored, but might be related to Opioid receptor in arcuate nucleus | Rabbits |

Notes: ^1^ Nomenclature and location of acupuncture points were referred to World Health Organization Western Pacific Region, WHO Standard Acupuncture Point Locations in the Western Pacific Region. 2010, Beijing, China: People's Medical Publishing House.

**References**

1. Li P, Tjen ALSC. Mechanism of the Inhibitory Effect of Electroacupuncture on Experimental Arrhythmias. *J Acupunct Meridian Stud* (2013) 6(2):69-81. Epub 2013/04/18. doi: 10.1016/j.jams.2012.11.001.

2. Magden ER, Sleeper MM, Buchl SJ, Jones RA, Thiele EJ, Wilkerson GK. Use of an Implantable Loop Recorder in a Chimpanzee (Pan Troglodytes) to Monitor Cardiac Arrhythmias and Assess the Effects of Acupuncture and Laser Therapy. *Comp Med* (2016) 66(1):52-8. Epub 2016/02/18.

3. Duan Y. The Different Antiarrhythmic Effects and the Mechanism of Electric Needle Stimulating Different Choujungs in Rats [Article in Chinese] [Master]: Hubei University of Chinese Medicine (2009).

4. Bian H. Effect of Ear Acupuncture of "Thoracopneumatic Area" on Ventricular Premature Comtraction Rabbits [Article in Chinese] [Master]: Kunming Medical University (2005).

5. Zhong H. Experimental Study on Inhibition of Neurogenic Arrhythmia by Electroacupuncture on "Neiguan" and "Shenmen" [Article in Chinese] [Master]: Beijing University of Chinese Medicine (2002).
